# Mr. Conrad W. Thies

**Published:** 1911-08-12

**Authors:** 


### MR. CONRAD W. THIES.

### A PERSONAL APPRECIATION BY AN OLD PUPIL.

It was in January 1896 that I first met Mr. Thies. I was then working for Dr. Barnardo, down in Stepney Causeway, as a correspondence clerk. I was sick of my job, not because I was badly treated but rather from envy of the gifts possessed by the brilliant Doctor and a realisation of my own poor powers as compared with those of my chief, as well as those of Mr. Adam Fowler, my immediate official superior, who had brought me up to town from Yorkshire. I used in those days to answer letters all day long, and I had something to do with the clerical work relating to the children's hospital which stands opposite to the offices of Dr. Barnardo's Homes and which is a part of that immense concern. Just as I went to Barnardo's Homes out of a crowd of unbefriended candidates, so I went to Thies. He came down to Stepney twice from Gray's Inn Road for the purpose of finding out all there was to find out about me. He told me that he had liked my letters of application for the post of steward and assistant secretary of the Royal Free Hospital. I remember that when he first called and found me in the midst of piles of letters he asked if he could have a chat with the man who was "over" me. Mr. Fowler was absent and I introduced Thies to the senior clerk, who was a good, plodding, painstaking chap, but a dull dog and in no way, in any circumstances, an inspired creature.

Thies had a long chat with this well-meaning man, and when he had done he returned to me and said: "Do you take your orders from him?" I replied: "Well, he thinks I do, but I admit no more!"

I was appointed Thies' assistant at the Royal Free in March 1896, and I worked with him for nearly eleven years. In leaving Barnardo for Thies I left a genius for a business man. Barnardo was an easy first in all charity circles. How he could read with staggering rapidity an important communication, think out the far-reaching answer, and dictate his reply I will not attempt to describe. How he could organise! How he could advertise! How he could get the place of top dog! He was deaf; he was violently impatient; he was mysterious.

Thies I found to be a very different man. For the first few months of my time I almost wondered if it could really be so difficult to run a big London hospital. But I was young. Month followed month and I began to open my eyes. Thies was a business man; he was a travelled man; a man of wide and wonderful reading powers; a judge of character; a keen chess-player; a strong cyclist; a man who moved things just as he desired they should be moved. In my inexperience I used to wonder why he had not become a merchant prince, for I thought his English was more like that of the counting-house than that of the adviser to, and the writer for, a Hospital Committee. Yes, I was young; I did not know Thies as I knew him later.

While working for Dr. Barnardo I had lived at Balliol House, Toynbee Hall, Whitechapel. Thies had discovered that, and he had gone to the trouble of calling upon Canon Barnett, the Warden of Toynbee, and putting question after question as to my private character and reputation, my habits of life, and so on. He wanted a man as his assistant at the Royal Free who would assist him; so he blandly put the matter, and he left no stone unturned, so far as he could judge, to get just the sort of man he knew he wanted. I found that Thies knew Whitechapel as well as I did, and I had lived in the heart of the district for three years. I found that it was Thies who had been largely instrumental in founding, the excellent programmes of music on Sunday evenings at South Place, Finsbury. I found he knew such men as Oscar Wilde and George Bernard Shaw. Then I began to admit that in leaving Barnardo I had gone to a man who was of a totally different mental mould, but one from whom I ought to learn much.

I attribute Thies' unquestioned and outstanding success as a hospital secretary to his business abilities coupled with his extraordinary powers of observation. Successes as hospital secretaries are by no means common. I know more than one chairman who has to redraft almost the whole of the secretary's annual report. Thies was a success from the first. He knew how to make his clerks work; how to rehearse a board meeting; how to enlist the practical interest of a casual visitor; how to pull round a wavering subscriber; how to deal with a wayward architect; he knew the powers and the weaknesses of a big and influential medical staff; he knew when to work and how to work and when work paid and when it did not. He was always watching and waiting; he knew as well as I did when I had "put in" a good morning's work; whether I was keeping the eighteen or twenty male servants in hand; he knew when he walked swiftly through the outer office if all was well. A glance was an inspection.

He had a marvellous memory for names and family associations. He would eat his lunch within sight of his desk much too quickly, reading the deaths in the "Times" as he swallowed his food! His luncheon over, he was correcting his list of subscribers, removing the names of any persons whose decease he had just read of.

And Thies in his pleasant home overlooking the fields and the lanes and the hedgerows at farther Finchley! How he loved his garden, his greenhouse, and his dog! How many local interests he had. He gave me many happy hours when I was without many friends, and in his home he compelled me—his assistant—to be his equal and his familiar friend, his brother and his companion on cycling runs, his fellow gardener and partner at the card table. His wide sympathies widened in his own happy home and his affection created affection.

Who will follow Thies? There are good men looking out, but there will not be another secretary at the Royal Free just like Thies, who goes by-and-by into retirement because he "feels there comes a time when a man should make way for others, and not wait until he is quite unfitted to obtain any satisfaction from leisure." Those quoted words are not mine; they were written by Thies and they bring a smile to my face, for I know that leisure and Thies are very far apart. When he is on holiday he is too busily observing, thinking, and planning and helping somebody with something. May he live long, and learn to love and be satisfied with his well-earned and richly-deserved leisure! C. C.

